# Supplementary material for: The SAGA HAT module is tethered by its SWIRM domain and modulates activity of the SAGA DUB module
Source: Biochim Biophys Acta Gene Regul Mech. Author manuscript; Available in PMC 2023 Jun 1. (PMC10226619; doi:10.1016/j.bbagrm.2023.194929)
Supplement: MMC1 [file NIHMS1892264-supplement-MMC1.docx]

**Supplementary Information**


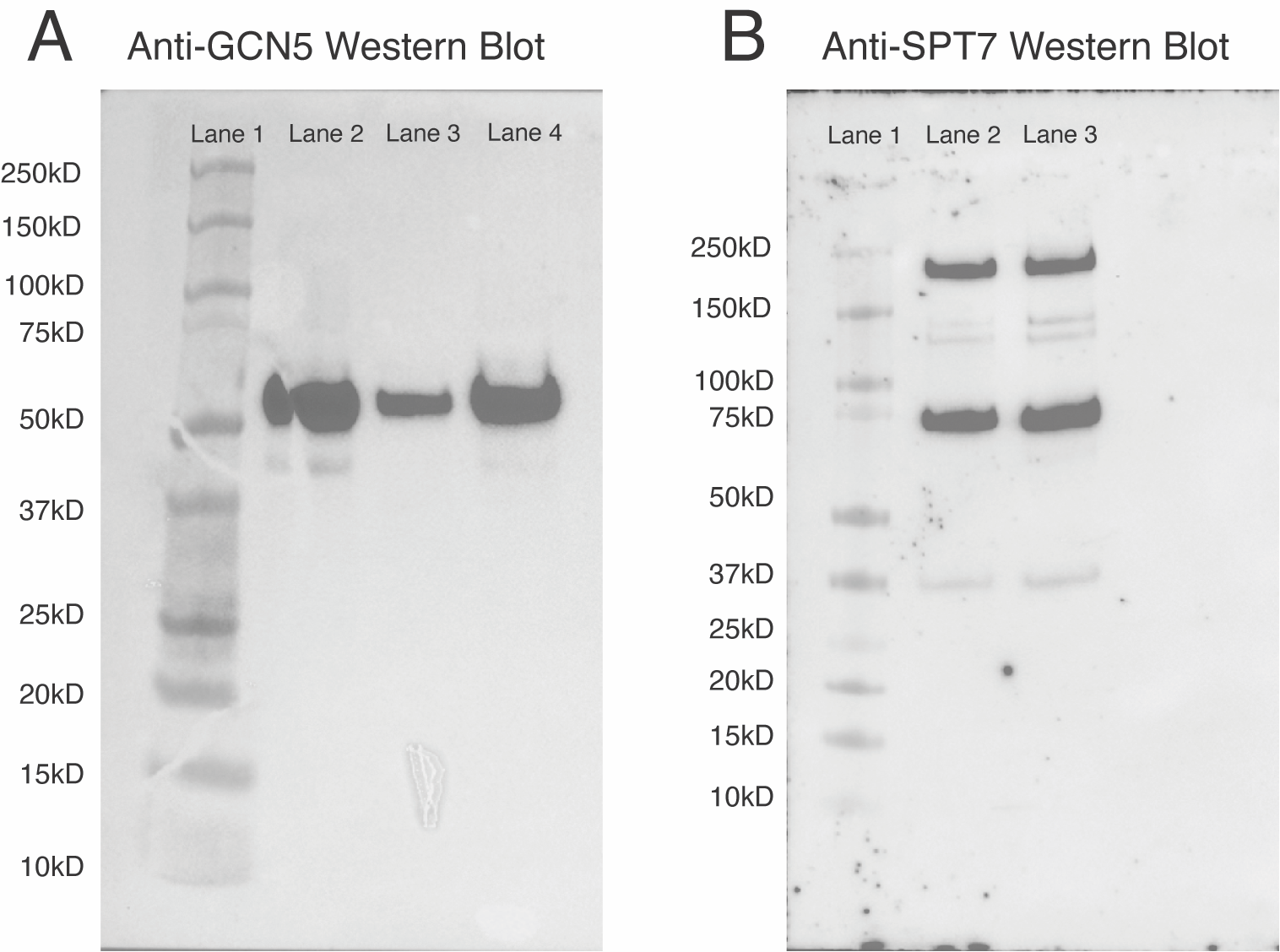


**Supplementary Figure S1:** Western blot visualization of WT SAGA and ∆SWIRM SAGA complexes. A: Anti-GCN5 western blot with lanes (1) Ladder, (2) WT SAGA (81nM), (3) ∆SWIRM SAGA (81nM), and (4) 7X ∆SWIRM SAGA (568nM). E: Anti-SPT7 western blot using an anti-TAP antibody with lanes (1) ladder (2) WT SAGA (81nM), and (3) ∆SWIRM SAGA (81nM).


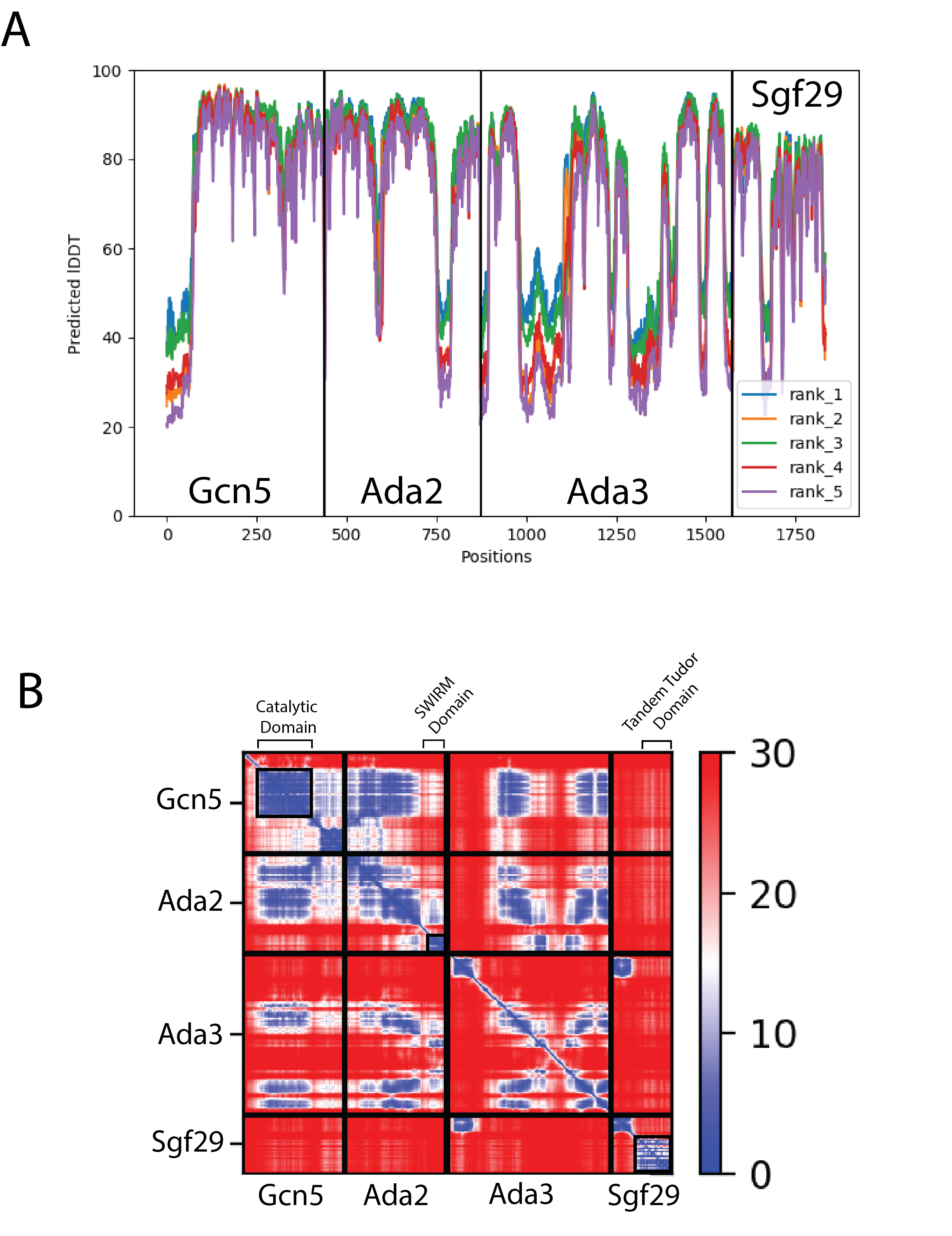


**Supplementary Figure S2.** AF2-Multimer model accuracy metrics. **A)** Predicted Local Distance Difference Test (pLDDT) score of the top five predicted structures of the HAT module. **B)** Predicted Alignment Error (PAE) score of the highest rank model. Areas corresponding to the SWIRM, tandem Tudor, and the catalytic domain are marked.


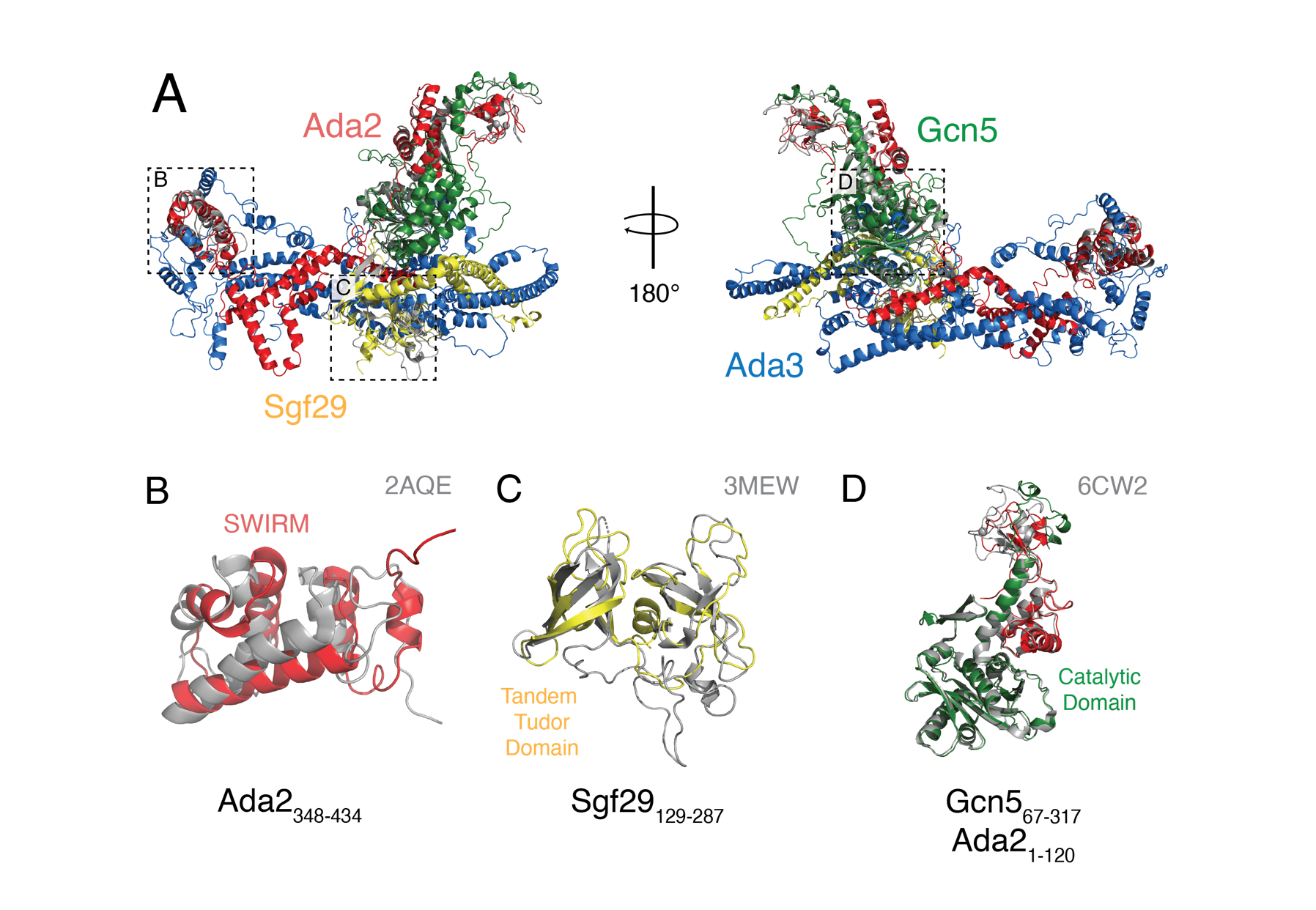


**Supplementary Figure S3.** Comparison of AF2-Multimer model of yeast HAT module against experimentally determined structures of HAT module components. (A) Overview of HAT module with crystal structures superimposed in gray. Boxes with dotted lines indicate portions of structure shown in panels B, C, and D. (B) Structure alignment of the solution structure of Ada2’s SWIRM domain (2AQE, gray) and the AF2-Multimer model (red). (C) Structure alignment of the crystal structure of Sgf29’s tandem Tudor domain (3MEW, gray) and the AF2-Multimer model (yellow). (D) Structure alignment of the crystal structure of Ada2_1-120_ and Gcn5_67-317_ (6CW2, gray) and the AF2-Multimer model (green and red).
